# Stress Exposures and Cognitive Health Trajectories in Later Life: The Moderating Role of Religious Involvement

**DOI:** 10.1093/geroni/igaf122.3657

**Published:** 2025-12-31

**Authors:** Xi Zhu

**Affiliations:** Oregon State University, Corvallis, Oregon, United States

## Abstract

A growing body of research has identified stress as a significant factor influencing cognitive health in later life. However, it remains unclear how stress exposures impact the rate of cognitive decline over time. As an important part of life for many older adults, religion provides a unique set of psychosocial resources that may function as a stress buffer. Yet, little research has directly examined the role of religious coping in the stress-cognition link. Using data from the Health and Retirement Study (HRS; 2006-2018), this study examines the effects of four types of stress exposures (lifetime stressful events, cumulative chronic stress, financial strain, and everyday discrimination) on cognitive health trajectories among older adults. It also investigates whether religious involvement moderates the potential adverse effects of stress on cognition. Results from growth curve models show that high levels of chronic stress, financial strain and everyday discrimination were associated with poorer cognitive function at baseline, whereas the total number of lifetime stressful events was positively related to baseline cognition. None of the stress exposures was associated with the rate of cognitive decline over time. In addition, religious attendance and perceived religious importance significantly mitigated the negative impact of financial strain and discrimination on baseline cognitive function. This study contributes to a deeper understanding of the relationship between stress, religion, and cognitive health. It highlights the potential protective role of religious involvement in promoting cognitive resilience against stress in the aging population.

